# Are muscle synergies useful for neural control?

**DOI:** 10.3389/fncom.2013.00019

**Published:** 2013-03-21

**Authors:** Aymar de Rugy, Gerald E. Loeb, Timothy J. Carroll

**Affiliations:** ^1^Centre for Sensorimotor Neuroscience, School of Human Movement Studies, The University of QueenslandBrisbane, QLD, Australia; ^2^Department of Biomedical Engineering, University of Southern CaliforniaLos Angeles, CA, USA

**Keywords:** aiming movement, muscle coordination, motor control, biomechanics, optimal control

## Abstract

The observation that the activity of multiple muscles can be well approximated by a few linear synergies is viewed by some as a sign that such low-dimensional modules constitute a key component of the neural control system. Here, we argue that the usefulness of muscle synergies as a control principle should be evaluated in terms of errors produced not only in muscle space, but also in task space. We used data from a force-aiming task in two dimensions at the wrist, using an electromyograms (EMG)-driven virtual biomechanics technique that overcomes typical errors in predicting force from recorded EMG, to illustrate through simulation how synergy decomposition inevitably introduces substantial task space errors. Then, we computed the optimal pattern of muscle activation that minimizes summed-squared muscle activities, and demonstrated that synergy decomposition produced similar results on real and simulated data. We further assessed the influence of synergy decomposition on aiming errors (AEs) in a more redundant system, using the optimal muscle pattern computed for the elbow-joint complex (i.e., 13 muscles acting in two dimensions). Because EMG records are typically not available from all contributing muscles, we also explored reconstructions from incomplete sets of muscles. The redundancy of a given set of muscles had opposite effects on the goodness of muscle reconstruction and on task achievement; higher redundancy is associated with better EMG approximation (lower residuals), but with higher AEs. Finally, we showed that the number of synergies required to approximate the optimal muscle pattern for an arbitrary biomechanical system increases with task-space dimensionality, which indicates that the capacity of synergy decomposition to explain behavior depends critically on the scope of the original database. These results have implications regarding the viability of muscle synergy as a putative neural control mechanism, and also as a control algorithm to restore movements.

## Introduction

There is now considerable evidence from a broad range of tasks and contexts that the activity of multiple muscles can appear to be well-approximated by only a few muscle synergies, each defined as a set of fixed relative levels of muscle activation (d'Avella et al., 2003, 2006; Torres-Oviedo et al., 2006; Tresch and Jarc, 2009; Dominici et al., 2011; Roh et al., 2012). Although many view this as a sign that muscle synergy is an important principle used by the nervous system to control movement, we believe that the viability of synergies as control elements needs to be evaluated in relation to task achievement rather than only to accuracy in accounting for observed muscle activity. All synergy decomposition procedures (including, for example, those based on convenient optimization algorithms such as the non-negative matrix factorization; Lee and Seung, 2001), care only about explaining as much variance of muscle activity as possible. These procedures are therefore completely blind to any consideration of task achievement, ignoring the functional significance of the (typically modest) muscle-space errors that are inevitably introduced when approximating an original muscle pattern using fewer synergies than muscles. However, because the musculoskeletal system has complex and highly non-linear properties, statistical methods that minimize errors in the input signal (muscle activity) may result in unacceptably large errors in the output (limb kinematics). A careful assessment of behavioral errors introduced by synergy decomposition therefore appears necessary to evaluate the viability of synergy as a potential biological control principle. Such errors would also affect the utility of synergy decomposition as a potential control strategy to restore movement artificially such as with functional electrical stimulation (FES) or myoelectric controls (Davoodi et al., 2003; Parker et al., 2006; Hargrove et al., 2009).

Neptune and colleagues evaluated whether muscle synergies extracted during human walking would actually produce well coordinated locomotion (Neptune et al., 2009; Allen and Neptune, 2012). They found that the activations of muscle synergies required substantial fine-tuning based on their consequences in task-space (i.e., minimizing difference between actual and simulated walking kinematics and ground reaction forces) to achieve satisfactory motor behavior. This suggests that relatively small errors produced by muscle synergies in reproducing muscle activation patterns can lead to important functional deficits. Other studies demonstrated the capacity to generate functional movements with a limited number of muscle synergies (McKay and Ting, 2008, 2012; Berniker et al., 2009; Kargo et al., 2010). These also required fine-tuning of synergy activation to produce reasonable behavior, a requirement that might result from discrepancies between the real and modeled biomechanics. None evaluated the functional consequence of synergy decomposition by comparing the movements predicted by the extracted synergies with those actually occurring when the basis-set of electromyograms (EMG) signals was recorded.

A likely reason for the lack of attention that has been devoted to the functional consequences of synergy approximation is the complexity of the mapping between muscle activities and their resulting effects on the limb. In addition to an accurate biomechanical model, effective forward simulation of limb kinematics from EMG requires an accurate measurement of the activation of each muscle. However, EMG are subject to crosstalk (i.e., contamination by nearby muscles) and representativeness issues (e.g., regional segregation of early recruited, slow-twitch motor units vs. late-recruited, fast-twitch units; Chanaud et al., 1991b), and are therefore imperfect measures of muscle activation (Staudenmann et al., 2010; Hug, 2011). To accommodate for this, we designed a practical forward simulation approach whereby a virtual representation of muscle biomechanics is defined that best reconstructs force when driven by EMG recordings (de Rugy et al., 2012c). This “virtual biomechanics” technique offers a unique opportunity to map the functional consequences of synergy approximation: Because the mapping between muscle and force is explicitly defined and used to control the task, the mapping between muscle synergies and force is also explicit and enables unambiguous assessment of the functional consequences of using extracted synergies compared to the original EMG signals in order to account for the motor behavior actually measured during those EMG recordings.

Another advantage of the explicit representation of muscle biomechanics defined with this technique is that it provides an experimental basis from which we can compute muscle activity according to the principles of optimal control theory; to achieve the task while minimizing a cost such as effort or variability of movement. Optimal control theory has been shown to reproduce patterns of muscle recruitment that are consistent with the existence of motor synergies (Todorov, 2004; Chhabra and Jacobs, 2006; Diedrichsen et al., 2010), and muscle synergies have been used to simplify the computational cost of optimization in several optimal control schemes (Todorov et al., 2005; Lockhart and Ting, 2007; Berniker et al., 2009). Here, we compared directly synergy decomposition and the resulting task performance between real data and simulated optimal muscle patterns for the same task. This comparison should indicate whether synergies similar to those extracted from real data can result from an optimal control scheme, and whether we can use simulated optimal muscle patterns in place of unavailable EMG to explore functional consequences of synergy decomposition in arbitrary biomechanical systems. First, we evaluated the functional consequences of synergy decomposition using previous data obtained when subjects performed force-aiming in two dimensions at the wrist, with force reconstructed online from EMG recordings (de Rugy et al., 2012c). Then, we computed the optimal muscle pattern that minimized the summed squared muscle activities (Fagg et al., 2002; Diedrichsen et al., 2010) for the virtual biomechanics extracted for each subject, and compared synergy decomposition on this simulated pattern with that obtained on real data. We also assessed synergy decomposition on the optimal muscle pattern for the higher dimensional elbow-joint complex (13 muscles), and explored reconstruction from an incomplete set of muscles as this represents the vast majority of cases for which recordings are available from only a subset of contributing muscles. The idea that sampling of muscle degrades the estimate of muscle synergies has been addressed in the literature (Clark et al., 2010; Ting and Chvatal, 2010; Allen and Neptune, 2012), but here we additionally determined the influence of the redundancy of the muscle selection on both synergy approximation and task performance. Finally, we explored the implications of increasing the dimensionality of the task (i.e., from 2-d to 3-d) for synergy decomposition of the optimal muscle pattern for an arbitrary biomechanical system. The scope of the original database is known to influence the results of synergy decomposition (Macpherson, 1991; Ting and Chvatal, 2010; Burkholder and van Antwerp, 2012), and we wanted to evaluate the influence of task dimension on synergy decomposition of optimal muscle patterns.

## Materials and methods

### Wrist experiment

We re-analyzed data from Experiment 1 in de Rugy et al. (2012c), applying synergy decomposition methods and measures, and additionally assessed their consequences in task space.

#### Participants

Six healthy, right-handed subjects (all men, aged 23–38) volunteered for this study. All had normal or corrected to normal vision and gave informed consent prior to the experiment, which was approved by the local ethics committee and conformed to the Declaration of Helsinki.

#### General procedure

**Figure 1:**
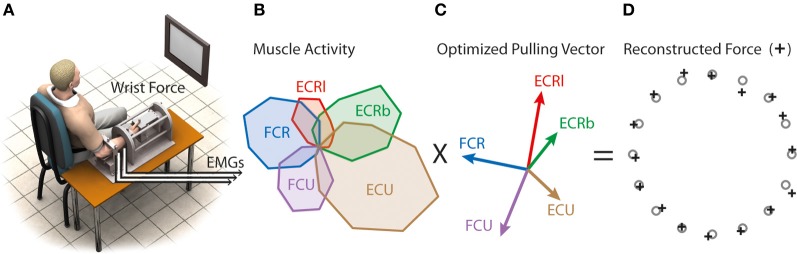
**Virtual biomechanics. (A)** Subjects produced force at the wrist to 16 targets. **(B)** Example of muscle tuning curves obtained by averaging EMGs from five trials per target in the initial force-driven task. **(C)** Virtual muscle pulling vector optimized to produce the best aiming performance when combined with muscle activity. **(D)** Aiming force reconstructed by combining **(B)** and **(C)**.

Subjects sat 80 cm from a computer display positioned at eye level. The right hand was maintained in a custom-made manipulandum with the forearm in a neutral position (midway between pronation and supination, as displayed Figure 1). The elbow was kept at 110° with the forearm parallel to the table and supported by a custom-built device. The wrist was fixed by an array of adjustable supports, contoured to fit the hand at the metacarpal-phalangeal joints (12 contacts) and the wrist just proximal to the radial head (10 contacts). This allowed wrist forces to be applied without the need for a gripping force. Wrist forces were recorded using a 6-df force/torque transducer (JR3 45E15A-I63-A 400N60S, Woodland, CA) coupled with the wrist manipulandum.

Real-time visual feedback of either the real wrist forces or the reconstructed wrist forces was presented on the visual display. Targets were presented at 16 radial positions around the center of the display (i.e., 22.5° apart). Flexion/extension corresponded to the horizontal axis (flexion left) and radial/ulnar deviation corresponded to the vertical axis (radial deviation up).

A block of 32 maximal voluntary contraction (MVC) trials was first conducted for each subject. This block was used to normalize the activity of each muscle during the aiming task to the maximal EMG obtained in that muscle during MVC toward any target direction. Each of the 16 target directions was presented twice in a randomized order. For each direction, subjects were asked to raise their force rapidly to the maximal extent while maintaining the force direction within a delineated range of ±8° of target direction. Maximal forces were held for approximately 2 s. Fifteen seconds were allowed for rest before the next target appeared in another direction.

The experiment contained a “force-driven” block in which the visual cursor used to reach targets represented the real force, followed by an “EMG-driven” block in which the cursor represented the reconstructed force. The force-driven block consisted of 96 trials (six trials for each of the 16 target directions) in which a low level of force (i.e., 22.5 N, which represents approximately 20% MVC for the subjects tested) was required to reach targets. This level of force was identical across all subjects, and chosen to reduce the possibility of fatigue. Each trial began only if the cursor was maintained less than 5% of the target distance from the origin continuously for 200 ms. The origin was calibrated to zero force along both axes (wrist relaxed) prior to each block. A random delay (1–2 s) elapsed before a single target appeared coincident with a brief tone. Participants were asked to move the cursor to the target with a movement time of between 150 and 250 ms, defined as the time between 10 and 90% of the radial distance to the target, and to hold the cursor continuously for 1 s within the target zone (a trapezoid ±8° from target direction by 10% of radial distance to target). A high-pitched tone signaled that the target had been acquired. If the target was not acquired within 2 s of target presentation, a low-pitched tone indicated the end of the trial. A second tone (200 ms after the first) indicated whether the movement time was correct (high tone) or not (low tone), and a bar graph provided visual feedback of the movement time in relation to the prescribed time window. Both the target and cursor disappeared at target acquisition or trial end, and at least 1 s elapsed before the start of the next trial. For each block, six consecutive trials were conducted for each one of 16 randomly ordered targets. The “EMG-driven” block was identical to the “force-driven” blocks, with the only exception that the real force feedback was replaced by the reconstructed force.

#### EMG procedure

Bipolar electromyographic signals were recorded from extensor carpi radialis longus (ECRl), extensor carpi radialis brevis (ECRb), flexor carpi radialis (FCR), flexor carpi ulnaris (FCU), and extensor carpi ulnaris (ECU) muscles, with self-adhesive surface electrodes. Signals were band-pass filtered from 30 Hz–1 KHz, amplified 200–5000 times (Grass P511, Grass Instruments, AstroMed, West Warwick, RI, USA), and sampled at 2 KHz. Electrode locations were determined according to procedures previously reported (Selvanayagam et al., 2011).

#### Data reduction and analysis

Muscle tuning curves, or the time-independent muscular activity (**a**) for the different target directions, were determined for each muscle as the mean rectified EMG during the hold-phase of the task (i.e., in a time window from 300 to 1000 ms after movement onset), averaged over five trials to each target (the first of the six consecutive trials to each target was discarded to prevent the uncertainty about target direction from contaminating the data).

Virtual biomechanics, the representation of muscle biomechanics that best reaches the target when combined with EMG data, was extracted from muscle tuning curves obtained from the “force-driven” block as indicated in de Rugy et al. (2012c). A coordinate descent was used to determine the set of pulling vectors **P** (Figure 1) that resulted in the best aiming performance, i.e., that minimizes endpoint errors *E* = ‖**x**_targ_ − **x**‖^2^ between cursor positions **x** (**x** = **P a**) and target positions **x**_targ_. This coordinate descent used the following steps: (1) Assign random values to the initial set of pulling vectors in the physiological range of muscle force and direction. (2) Pick a muscle at random and modify its pulling vector by changing its endpoint by a step in four orthogonal directions. The target errors associated with each of the five pulling vectors (i.e., the original and the four modified for that muscle) was then calculated as the summed squared error between targets and reconstructed reaches, and the pulling vector that produced the lowest cost was retained. (3) One iteration of the model was said to be completed when each muscle had been optimized once. (4) The whole model was iterated until the overall cost converged to a low value.

The resulting set of pulling vectors was then multiplied online by the rectified filtered EMG of the five muscles to reconstruct force used as a feedback in the “EMG-driven” condition. This virtual biomechanics was also used to compute the optimal muscle pattern, as explained below. We showed previously that muscle tuning curves for this data set were not different in the “force-driven” and in the “EMG-driven” blocks (de Rugy et al., 2012c).

Synergy extraction was conducted using the non-negative matrix factorization algorithm (Lee and Seung, 2001) on muscle tuning curves obtained in the “EMG-driven” block, for which we unambiguously know the mapping between muscle activity and task space. The muscle activation pattern **a** was first normalized such that each muscle has unit variance, and the normalized pattern a^*^ was approximated with N muscle synergies according to

$$a^{*}\approx {\hat{a}}^{*}=c\;w$$

where a^*^ is a matrix with each component representing the normalized activation of a specific muscle for a specific target direction, â^*^ is the approximated muscle pattern, **w** is a matrix with each component representing the activation of a specific synergy for a specific target direction, and **c** is a matrix of non-negative scaling coefficients.

The goodness of synergy approximation was calculated as a multivariate R^2^ (Mardia et al., 1979; d'Avella et al., 2006):

$${\text{R}}^{2}=1-\frac{SSE}{SST}=1-\frac{{\left\|a^{*}-{\hat{a}}^{*}\right\|}^{2}}{{\left\|a^{*}-\;{\bar{a}}^{*}\right\|}^{2}}$$

where *SSE* is the sum squared residuals and *SST* is the summed squared residual from the mean normalized activation vector (ā^*^). We also computed the variance accounted for (VAF), a related measure where *SST* is simply the summed squared activation, i.e., calculated on uncentered data (Cheung et al., 2005; Roh et al., 2012):

$$\text{VAF }=\;1-\;\frac{SSE}{SST}=1-\;\frac{{\left\|a^{*}-\;{\hat{a}}^{*}\right\|}^{2}}{{\left\|a^{*}\right\|}^{2}}$$

For each synergy decomposition, an associated aiming error (AE) was computed as the distance between targets **x**_targ_ and the force vector produced by combining the pulling vectors with the (un-normalized) muscle activity approximated by the synergies $\left(\hat{x}=P\hat{a}\right)$:

$$\text{AE }=\;{\left\|\hat{x}-\;x_{\text{targ}}\right\|}^{2}$$

For each subject and synergy number (*N* = 1 − 5), the synergy decomposition was conducted 10 times and averaged values were obtained for each of the three measures (R^**2**^, VAF, and AE).

### Wrist simulations

We computed the optimal muscle pattern **a**_opt_ for the set of pulling vectors extracted individually for each subject using the procedure described in Fagg et al. (2002). This procedure minimizes the following composite cost *C*, which ensures task achievement by minimizing target errors while simultaneously minimizing the summed squared muscle activations:

$$C=\frac{1}{2}{\left\|x_{\text{targ}}-x\right\|}^{2}+\frac{\lambda }{2}{\left\|a_{\text{opt}}\right\|}^{2}$$

where λ is a regularization parameter set to 0.02 to represent allowable errors on the order of 2% of movement magnitude. Synergy decomposition was applied on the optimal muscle pattern as on experimental data, and differences between experimental and simulated data were tested using a two way [data type (experimental vs. simulated) × number of synergy (1–5)] repeated measures ANOVAs for the three measures (R^2^, VAF, and AE). Differences between AEs produced with different number of synergies were also tested on experimental data using a paired sample *t*-test. The significance level was set to α = 0.05.

### Elbow simulations

The optimal muscle pattern was also computed on an existing biomechanical model of the arm for a similar center-out isometric task performed at the elbow joint complex in the flexion/extension and supination/pronation workspace (de Rugy et al., 2009; de Rugy, 2010). The biomechanical model developed by Davoodi and colleagues (Figure 4A; Davoodi et al., 2002a,b; de Rugy et al., 2008; de Rugy, 2010) was used to extract the pulling vectors of 13 arm and forearm muscles in this workspace (Figure 4B): Supinator (SUP), the short and long heads of Biceps Brachii (BIC ln and sh), Brachialis (BRA), Brachioradialis (BRD), Pronator Teres (PT), Pronator Quadratus (PQ), the long, medial, and lateral heads of Triceps (TRI ln, m, and lt), and three wrist muscles (FCR, ECRl, and ECRb; please note that the two remaining wrist muscles, FCU, and ECU, were not included because their moments are negligible in that workspace).

Synergy decomposition was conducted on the optimal muscle pattern according to the method described above for the wrist, and the procedure was repeated 100 times to obtain the mean and standard error of the three measures (R^2^, VAF, and AE).

To account for the vast majority of cases in which recordings are limited to an incomplete set of muscles, we also explored reconstructions from a limited number of muscles. In particular, we considered two qualitatively different selections of eight muscles amongst the 13 muscles: a “redundant” selection (Figure 5A, BIC ln and sh, BRA, BRD, TRI ln, lt, and m, PT), and a “less redundant” one (Figure 5B, BICln, BRA, TRIln, PT, SUP, PQ, FCR, ECRb). The idea behind this choice of qualitatively different sets of muscles was that the level of biomechanical redundancy might translate into relationships between activation of muscles that would be visible through synergy decomposition. R^2^ and VAF were calculated as before for these incomplete sets of muscles, and AE was computed on the basis of the virtual biomechanics reconstructed from these eight muscles only (i.e., based on the set of pulling vectors that best achieve targets when combining the activity of the eight muscles rather than on the true pulling vectors). This was designed to assess the quality of the reconstruction in both muscle space and task space for common situations in which recordings are only available from fewer muscles than those contributing the task.

### Simulations with arbitrary pulling vectors in 3 and 2 dimensions

We explored the implications of changing the dimensionality of the task-space for synergy decomposition using the optimal muscle pattern for an arbitrary biomechanical system represented by a set of 13 pulling vectors in three and two dimensions. A set of 13 unit vectors approximately uniformly distributed in three dimensions was first defined using a repulsive iterative algorithm (Figure 6A). Then the same iterative algorithm was used to generate a set of 200 targets approximately uniformly distributed on a sphere (Figure 6B), and the optimal muscle pattern for these pulling vectors and targets was determined that minimized the same cost function as for the wrist and elbow (i.e., composite cost with target errors and summed squared muscle activations). The corresponding simulations in two dimensions were conducted using the original set of 16 targets used for the wrist and elbow, and the same set of 13, 3-dimensional pulling vectors by ignoring the third dimension (Z on Figure 6B, 2-d pulling vector shown Figure 6C). The optimal muscle pattern was computed as before (Figure 6D). Synergy decomposition and the mean and standard error of the three main measures (R^2^, VAF, and AE) were conducted and obtained as for the elbow system.

## Results

### Synergy decomposition on real and optimal data at the wrist

**Figure 2:**
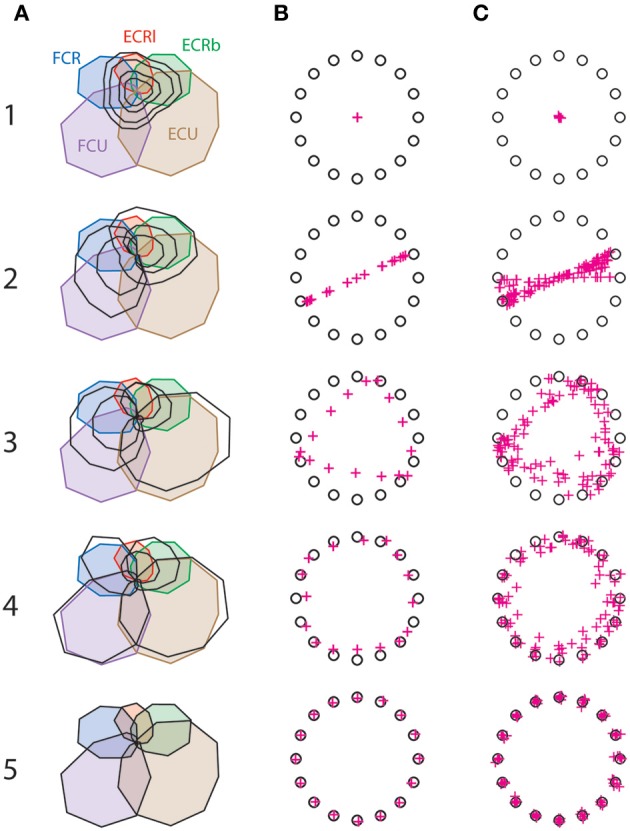
**Synergy decomposition at the wrist. (A)** Actual muscle pattern (colored) with the muscle pattern reconstructed with 1–5 synergies (black) for a representative subject. **(B)** Corresponding targets (circles) and reconstructed aiming force (crosses) for the same subject. **(C)** Targets (circles) and reconstructed aiming force (crosses) for all subjects.

Figure 2A shows how synergy decomposition approximates the original muscle pattern for one subject, and Figures 2B,C show how this approximation translates into AEs for the same subject (**B**) and for all six subjects (**C**). This figure illustrates that the muscle pattern reconstructed with synergy only starts to resemble the real pattern with three or four synergies, but also that the remaining muscle-space differences translate into substantial AE. These errors disappear only when five synergies have been extracted, which reflects the unreduced dimensionality of the complete musculoskeletal system.

**Figure 3:**
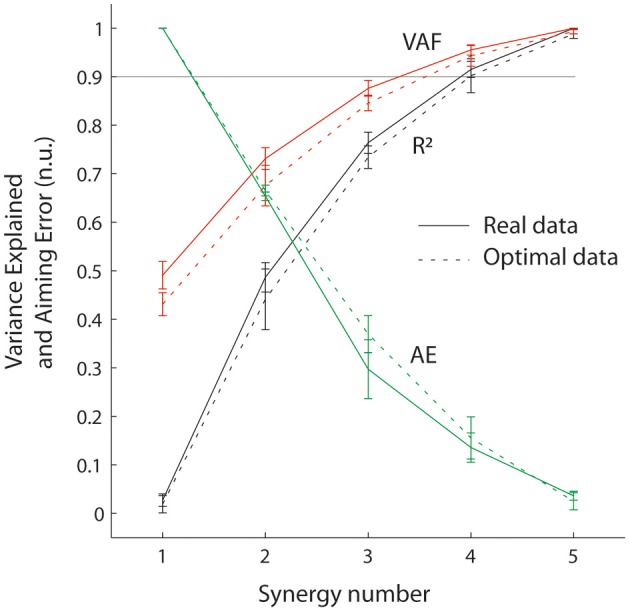
**Goodness of muscle and aiming force reconstructions at the wrist, evaluated through VAF, R^2^, and AE.** Sold lines correspond to real data and dotted lines correspond to simulated data (optimal muscle pattern). Error bars represent standard errors.

Figure 3 shows that the goodness of the muscle reconstruction increases as expected with the number of synergies, and that AEs decrease accordingly. It is worth noting that a VAF of approximately 0.5 is obtained with only one synergy, where the cursor hasn't moved from the center of the workspace toward any target (Figure 2C). This illustrates that 50% of the variance in muscle activity is accounted for by a synergy decomposition that is doing no better at reaching targets than simply not activating any muscles. Figures 2B,C also illustrate that synergy decomposition systematically results in undershoot errors. The reason for this is obvious with one synergy, where the solution found by the non-negative matrix factorization algorithm takes the form of muscles that co-contract to their average activity level in the original muscle pattern. For other number of synergies, the muscle pattern approximated with fewer synergies than muscles similarly results in wider muscle tuning curves, which produces undershoot errors when summing muscle contributions at the joint.

Figure 3 shows that the reconstruction with four synergies explained most of the variance of the muscle pattern (VAF = 0.95 and *R*^2^ = 0.91) while still producing considerable task errors (averaged error of 13.5 % of target distance, visible Figure 2C), that are significantly higher than errors produced by the original muscle pattern (3.6% averaged error; *t* = 8.11 *p* < 0.0005).

Figure 3 also shows that synergy decomposition conducted on optimal muscle patterns computed for the virtual biomechanics extracted from individual subjects generated similar results to synergy decomposition of real data. There were no differences in R^2^ or AE values calculated on real vs. simulated data [*F*_(1, 5)_ < 4.98, *p* > 0.08], although VAF values were slightly higher when calculated on real data than optimal data [*F*_(1, 5)_ = 9.19, *p* = 0.03]. These results indicate that synergy decomposition of optimal muscle recruitment patterns produces results similar to those obtained from real EMG signals. This makes it possible to explore the inherent consequences of synergies interacting with realistic musculoskeletal dynamics without the potential confounds introduced by missing, poorly sampled or noisy EMG signals.

### Synergy decomposition on optimal data at the elbow joint complex

**Figure 4:**
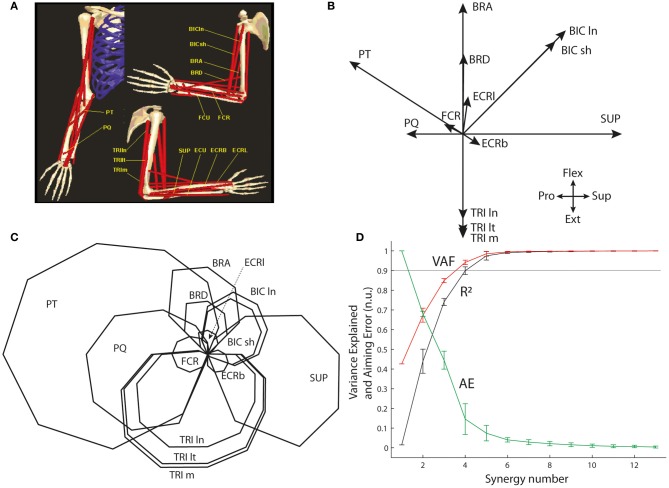
**Synergy decomposition at the elbow joint complex. (A)** Musculoskeletal model. **(B)** Pulling vectors of muscles in the two-dimensional space of flexion/extension and supination/pronation. **(C)** Optimal muscle pattern for this system. **(D)** Goodness of muscle and aiming force reconstructions, evaluated through VAF, R^2^, and AE.

Figure 4 shows that synergy decomposition conducted on the optimal muscle pattern (**C**) computed for the 13 muscles of the biomechanical arm model provides results (**D**) closer to those usually reported in the literature; i.e., a goodness of the reconstruction (both VAF and R^2^) that quickly rises to reach an asymptotic level at which most muscle variance is explained with substantially fewer synergies than muscles. For instance, the 90% variance level is reached with only four synergies (VAF = 0.94 and *R*^2^ = 0.90) and almost the entire variance is explained with five synergies (VAF = 0.98.5 and *R*^2^ = 0.97.5). However, the associated AEs are still substantial (14.6% and 7.5% of target distance with four and five synergies, respectively), and at least six synergies are needed to generate AEs below 5% of target distance (i.e., 4.1% with six synergies).

**Figure 5:**
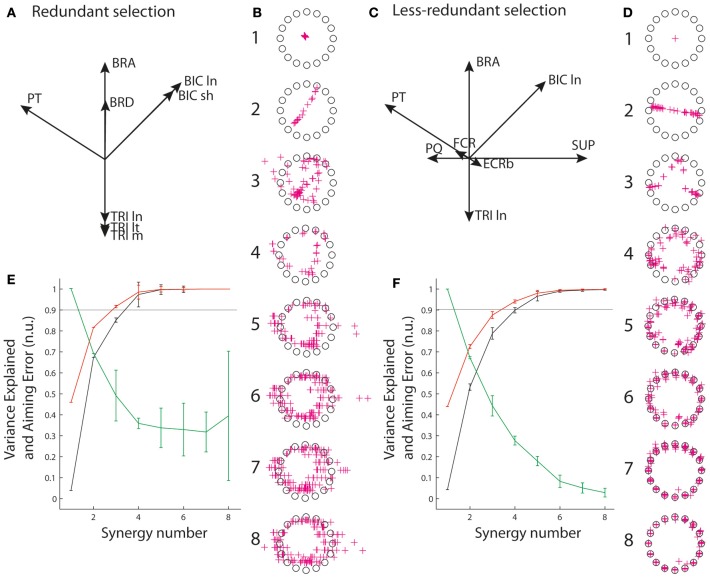
**Reconstruction from incomplete set of muscles. (A,C)** Muscle pulling vectors for the redundant **(A)** and less redundant **(D)** set of muscle. **(B,D)** Reconstructed aiming force. **(E,F)** goodness of muscle and aiming force reconstructions evaluated through VAF, R^2^, and AE.

The situation changes when only an incomplete selection of muscles is available to perform the synergy decomposition and the force reconstruction, reflecting the common experimental situation in which recordings are not available from all contributing muscles. Figure 5 shows that the goodness of muscle approximation is better for the selection of redundant muscles than for the selection of less redundant muscles. For instance, at least 90% of the variance is explained with three and four synergies with the set of redundant muscles, but this degree of variance explained requires an additional synergy with the set of less redundant muscles. Inversely, the AE reconstructed by combining synergy approximation with the virtual biomechanics extracted on the incomplete set of available muscles is much higher for the redundant muscles than for the less redundant muscles. In particular, the reduction of error saturates above 30% from 4 to 8 synergies for the set of redundant muscles, but monotonically decreases to a minimum of 3.8% for the less redundant muscles.

It is worth noting that if AEs associated with synergy decomposition are better for the set of less redundant muscles, they are still substantially higher than with the complete set of muscles. For instance, AEs for 5–7 synergies are 29, 17.5, and 8% for the set of less redundant muscles, and 14.6, 7.5, and 4.1% for the complete set of muscles. This indicates that aiming performance suffers more from synergy approximation with an incomplete set of muscles.

### Synergy decomposition on arbitrary biomechanics in 2 and 3 dimensions

**Figure 6:**
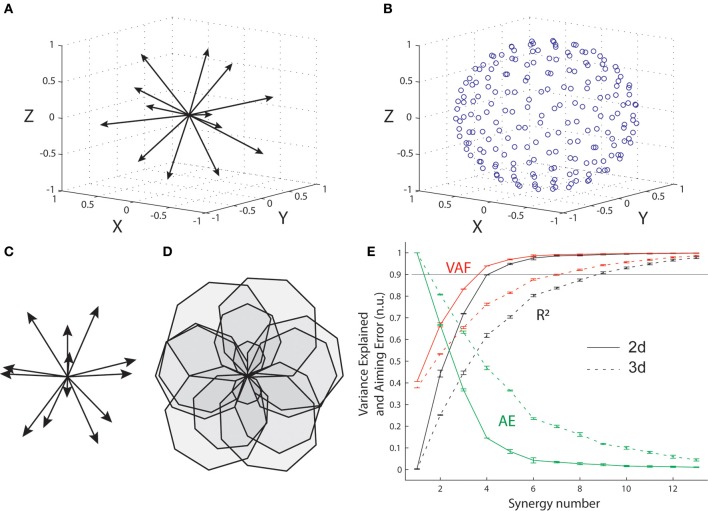
**Synergy decomposition from arbitrary muscle pulling vectors in 3-d and 2-d. (A)** Unit pulling vectors representing 13 muscles approximately uniformly distributed in the three-dimensional space. **(B)** Set of 200 approximately uniformly distributed targets on the surface of a sphere. **(C)** Corresponding pulling vectors in the two-dimensional space, obtained by suppressing the Z-dimension to the original set of 13 pulling vector. **(D)** Optimal muscle pattern in 2-d. **(E)** Goodness of muscle and aiming force reconstructions evaluated through VAF, R^2^, and AE, for both the 3-d and the 2-d simulated data sets.

Figure 6E shows that synergy decomposition conducted on the optimal muscle pattern computed on the 13 muscles of the arbitrary biomechanical model provides different results for the two- and three-dimensional versions of the task. As for the elbow system, the optimal muscle pattern for the 13 two-dimensional pulling vectors (Figures 6C,D) reaches the 90% variance level with only four synergies (VAF = 0.94, *R*^2^ = 0.90, AE = 14.6%). In contrast, nine synergies were required to reach the same 90% variance level for the three-dimensional version of the task (VAF = 0.94, *R*^2^ = 0.91, AE = 12%). For both cases, an additional synergy is required for the averaged AE to drop below 10% (i.e., 8% with five synergies and 9.8% with 10 synergies for the two- and three-dimensional cases, respectively). This indicates that the capacity of synergy to explain optimal muscle patterns and their functional outcome depends critically on the scope of the original database, where higher dimensional behaviors will require more synergies or will produce poorer fits.

## Discussion

The purpose of this study was to assess the functional consequences of approximating an actual pattern of muscle recruitment with fewer synergies. We first used previous data obtained when people performed a force-aiming task in two dimensions at the wrist, where force was reconstructed online from EMG recordings (de Rugy et al., 2012c), to show that despite successfully explaining muscle activities, synergy-approximated EMG data would introduce substantial errors in task space. Then, we showed that synergy decomposition on the optimal muscle pattern that minimizes summed-squared muscle activities for a representation of muscle biomechanics produces similar muscle approximations, with the same functional consequences. We also assessed the influence of synergy decomposition on the optimal muscle pattern computed for the more redundant elbow-joint complex, to show that when selecting an incomplete set of muscles, the redundancy of that selection has opposite effects on the goodness of muscle approximation and on task achievement: higher redundancy is associated with better muscle approximation, but with higher AEs. Finally, we showed that increasing the dimensionality of the task-space from 2 to 3 dimensions also increases the number of synergies required to approximate the optimal muscle pattern and produce low AEs.

If synergies are used only as a tool to summarize observed muscle activation patterns, then they are no different from regression analysis, in which the goodness of fit depends simply on the number of free parameters and the complexity of the source data. Instead, synergies were introduced originally by Bernstein (1967 translation of 1934 book) and continue to be offered as a theory for how the nervous system solves a very specific control problem known as redundancy. The musculoskeletal system contains more degrees of mechanical freedom and more muscles than required to perform tasks successfully. If the nervous system must compute the pattern of muscle activations required to perform a given task, how does it decide which of many solutions to use? One solution is to add performance criteria that can be optimized by one and only one solution (e.g., minimize trajectory errors in the face of noise or minimize effort to conserve energy). Computing or discovering such optimal solutions tends to be extremely difficult for systems with the complexity of a typical limb (reviewed by Valero-Cuevas et al., 2009 and Loeb, 2012). An alternative solution is for there to be arbitrary restrictions on the available patterns of muscle recruitment, either as a consequence of hard-wired neural circuits or learned motor habits. The synergies extracted by decomposition of observed EMG patterns would then be indicative of this control strategy at work. The validity of synergies as a neural control strategy thus depends on its necessity (what are the alternatives?) and its utility (what are the consequences?), which are discussed below.

### Muscle synergies introduce aiming errors

The importance of considering error introduced in the task space when assessing the usefulness of muscle synergies is clearly illustrated by the fact that in our wrist isometric task, about 50% of muscle variance is accounted for (i.e., VAF) by only one synergy. In this case, the solution found by the non-negative matrix factorization algorithm takes the form of muscles that co-contract to their average activity level in the original muscle pattern. When summing muscle contributions at the joint, this resulted in zero net force, which therefore translated in no movement whatsoever toward the force targets. This extreme case might indicate that centered data should be preferred when calculating the goodness of muscle approximation (i.e., use R^2^ instead of VAF) to avoid over-interpreting spurious results arising from the nature of the statistical method. However, both R^2^ and VAF are relatively insensitive to the functional consequences of the synergy approximation of muscle activity. For instance, approximating the activity of the five wrist muscles with four synergies explained most of the muscle variance (R^2^ and VAF > 0.9), while still missing the force targets by 13.5% of target distance. This is important because this order of magnitude of explained muscle variance has been considered as an accurate description in different contexts [e.g., R^2^ of 0.85 in reaching (Muceli et al., 2010) or VAF of 0.88 for locomotion (Oliveira et al., 2012)]. It is interesting to note that synergy decomposition systematically resulted in undershoot errors (Figure 2) because it inevitably produces inappropriate cocontraction.

The assessment of the functional consequences of muscle approximation by synergies was possible here because for the isometric task at relatively low force (approximately 20% of MVC), the relationship between muscle activity and task space is likely to be close to linear. Although we have not tested our virtual biomechanics technique in contexts where this might not be the case, this linear relationship might be required for subjects to perform the task similarly well with either the real force or the force reconstructed online from EMG recordings (de Rugy et al., 2012c). Because the synergy decomposition was conducted on data obtained when the task was performed with reconstructed force, the mapping between EMG and task space was known, and we applied this mapping directly to calculate errors introduced by synergy decomposition in task space. It remains that the mapping between muscle activity and task space is likely to be far more complex in broader dynamic contexts that include strongly non-linear relationships between muscle force and velocity for a given level of activation (Brown et al., 1999), which might introduce more errors. For example, the final positions of center-out reaches will depend more on the relatively small EMG signals but large forces that stop the movement on target than on the larger EMG signals but smaller forces in the agonists that accelerate the limb at the beginning of the task. This illustrates that an important part of the control problem might reside in an arbitrarily small proportion of unexplained muscle activation variance.

As mentioned in the introduction, we believe that the complexity of the mapping between muscle activity and the resulting action is the primary reason for the scant attention that has been devoted to the functional consequences of synergy approximation. Although Neptune and colleagues found that synergies were a useful starting point, they required substantial fine-tuning based on their consequences in task-space to produce well coordinated locomotion (Neptune et al., 2009; Allen and Neptune, 2012). There is no question that the statistical methods used to extract synergies from EMG recordings must capture substantial features of the very behavior from which the recordings were obtained. The question of whether these synergies reflect an organizing principle of neural control depends on their capacity to sufficiently account for behavior. This condition was not met in either the walking studies or in the wrist task presented here.

### Muscle synergies might arise from, or subserve optimal control

Despite the shortcomings mentioned above in relation to AEs generated with the wrist system, we found that synergy decomposition produces similar results on real and simulated (optimal) data. This direct comparison extends previous reports that optimal control schemes produce synergy-like properties (Todorov, 2004; Chhabra and Jacobs, 2006) by showing both qualitative and quantitative matches within the same protocol. Because the deleterious effects of synergy decomposition in terms of AEs at the wrist might relate to the relatively low muscle redundancy of that system, we also explored the more redundant elbow-joint complex. We show that synergy decomposition on the optimal pattern for the 13 muscles of that system produces results that correspond to those typically reported in muscle synergy studies (d'Avella et al., 2006, 2008; Roh et al., 2012), with a goodness of muscle approximation that quickly rises to reach an asymptote level at which most muscle variance is explained with substantially less synergies than muscles. Thus, properties that are typical of the muscle synergy hypothesis arise from the inherent principles of optimal control, highlighting the obvious possibility that synergy might just be a by-product of an alternate control scheme rather than a control principle in itself (Todorov, 2004; Chhabra and Jacobs, 2006; Diedrichsen et al., 2010). One such control scheme that could result in muscle activations that resemble the output of muscle synergies involves the online computation of optimal task solutions by feedback control laws (i.e., optimal feedback control; Todorov, 2004; Diedrichsen et al., 2010). However, muscle synergies have also been suggested to subserve optimal feedback control as a possible neural control element to reduce the high computational cost associated with online optimization (Todorov et al., 2005; Berniker et al., 2009) or to implement a simple feedback rule at task level (Lockhart and Ting, 2007; Ting and McKay, 2007).

Alternatively, muscle synergies might prevail over optimal control schemes. Similarities between real and optimal muscle patterns might reflect behaviors that have developed and evolved to minimize biologically relevant costs, and whose production is mediated by muscle synergies that are relatively less flexible at shorter time scale. In fact, we showed recently that when faced with novel biomechanics, participants adapted by scaling their original muscle patterns linearly rather than re-optimizing them (de Rugy et al., 2012b), which could at first glance appear in favor of the existence of hard wired synergies. However, we also found that muscle patterns observed when simulating the biomechanics of a posture different from the real posture were better described by a linear scaling of the muscle pattern associated with the real posture than with the simulated posture (de Rugy et al., 2012a,b). This result is not consistent with a re-optimization of activation signals to a set of fixed muscle synergies, as this should have enabled reproducing the optimal muscle pattern associated with the simulated posture. This result could potentially be explained by posture- or feedback-specific synergies (Cheung et al., 2005; d'Avella et al., 2008), but this would be inconsistent with the definition of muscle synergy as a fixed, linear combination of muscle activations, and the associated potential benefits in terms of dimensionality reduction for higher level controllers. Indeed, if synergies are allowed to vary depending on the task or context, these variations would become additional degrees of freedom requiring both additional circuits that can produce the additional synergies and control circuits to select among them. Alternatively, the observed limitation of flexibility of posture-dependent muscle patterns could conceivably pertain to stored and recalled activation signals to synergies rather than to the synergies themselves. However, this would be difficult to distinguish from alternative control schemes involving stored motor programs that would be based on an equal or higher number of control signals than muscles. The concept of synergies might be defined broadly to reflect any tendency to use muscles in learned patterns rather than requiring the existence of specific circuits that generate fixed combinations of muscle activations. But in that case, it is really a neologism for regression analysis that has no predictive value as a reductionist theory of motor control.

### The number of synergies increases with task dimension

Our simulations show that when going from aiming in two to three dimensions with the same (arbitrary) biomechanical system, more synergies are required to well-approximate the optimal muscle pattern and to generate sufficiently small errors (Figure 6E). This illustrates that the capacity of synergy to explain behaviors depends critically on the scope of the original database, where more diverse behaviors will require more synergies or will produce poorer fits. In other word, the seemingly high capacity to account for most activity of numerous muscles with only few linear synergies might hold in restricted experimental contexts (Loeb, 2000), but is expected to deteriorate for more diverse natural behaviors (Macpherson, 1991).

The need to study sufficiently rich behavioral sets has been well-recognized in EMG studies of behaving animals, in which it is possible to surgically implant selective and precisely positioned recording electrodes (Loeb and Gans, 1986). A monkey can learn gradually to selectively modulate muscles that appear to be closely synergistic on both anatomical and electrophysiological grounds if some mechanical advantage can thereby be attained (Cheng and Loeb, 2008). Cats walking on a treadmill exhibit stereotypical patterns of synergy in some major muscles but appear to have learned idiosyncratic patterns of use for smaller muscles (Loeb, 1993), which patterns depend on the musculoskeletal mechanics of the limb rather than genetically specified spinal pattern generators for locomotion (Loeb, 1999). Even within anatomically singular muscles, neuromuscular compartments that have somewhat different mechanical actions on the skeleton can be differentially recruited for some but not all tasks (Chanaud et al., 1991a,b; Pratt et al., 1991; Pratt and Loeb, 1991). These refinements of neural control are likely to go unappreciated in the EMG databases obtainable from surface electrodes, but they seem likely to be present nonetheless in humans.

### Implications for the use of synergy in artificial control

FES typically requires the transformation of a motor goal into control signals designed to stimulate muscles in order to achieve that goal (Davoodi et al., 2003; Loeb and Davoodi, 2005). We illustrated previously that the nervous system does not seem to re-optimize activation signals to a set of muscle synergies (de Rugy et al., 2012b), but this does not mean that the principle of low dimensional control modules cannot be useful to restore movements artificially with FES. For instance, optimal muscle patterns computed for the biomechanics of a particular limb could be decomposed into fewer synergies, that could be used to enable online optimization onto fewer control signals (Todorov et al., 2005), or to implement of simple feedback rules at task-level without requiring to solve complex redundancy problems (Lockhart and Ting, 2007; Ting and McKay, 2007). The value of such schemes, however, remains contingent upon whether their benefits in reducing computational cost outweigh task-space errors introduced by the original approximation into fewer synergies.

Myoelectric control is another important area where muscles are used to restore movement artificially, although in this case muscles are used to generate control signals rather than to receive them. In contrast to traditional myoelectric prostheses where muscle activities are translated into velocity about a joint, the goal of contemporary myoelectric control research is toward the simultaneous and proportional control of multiple degrees of freedom (Parker et al., 2006; Jiang et al., 2009). In this context, a recent technique that involves transferring residual nerves to alternative muscle sites targeted motor reinnervation (TMR) has increased the number of muscle signals available to control a prosthetic device in amputees (Kuiken et al., 2007, 2009). Although promising, this re-innervation technique is unlikely to restitute the complete set of original control signals. Similar recording limitations obtain in intact musculoskeletal systems. This is why we explored simulations with incomplete sets of muscles. The first consideration, before synergy decomposition, is to reconstruct the motor output from the available muscles. We showed previously that this could be done with our virtual biomechanics technique, which empirically finds a representation of muscle biomechanics that best reconstructs force when driven by EMG recordings (de Rugy et al., 2012c). Here, we additionally show that on simulated (optimal) muscle patterns, the reconstruction is better for an incomplete set of muscles that are less redundant than for a set of more redundant muscles. Then, irrespective of how good or bad this reconstruction is, synergy decomposition would add additional errors on the top of it. From that perspective, applying synergy decomposition onto the set of available muscles appears of little use, and it seems rather more appropriate to make full use of all available muscles to best reconstruct the motor output in task space. This is essentially what is now done with the method of principal components analysis of TMR recordings (Jiang et al., 2009).

Nevertheless, synergy decomposition might suggest a useful, although paradoxical way to decide which muscles to record from in the case of myoelectric controllers in which the number of available myoelectric channels is limited. Those muscles that are most difficult to decompose into a small number of synergies should be accorded the highest priority for obtaining command signals.

### A high dimensional alternative to synergy

Finally, we ask whether there really is a redundancy problem to be solved at all. The notion that the nervous system might control movement through a limited number of synergies seems at odds with the high number of neurons available to process the transformation between sensory information and motor commands, as well as with the numerous divergent pathways that have been suggested by some as a possible basis for the implementation of muscle synergies. For instance, the processes of sensorimotor transformation and adaptation are well described by gain fields, or population codes formed by numerous basis neurons each responding to a particular range or combination of inputs (Andersen et al., 1985; Salinas and Abbott, 1995; Pouget and Snyder, 2000; Baraduc et al., 2001). Although decoding algorithms such as those developed for neural prosthetics involve a great deal of dimensionality reduction to extract motor goals from neuronal populations (Musallam et al., 2004; Hauschild et al., 2012), there seems to be no compelling reason to believe that the nervous system should operate a comparable dimensionality reduction into muscle synergies before increasing the dimensionality again to pools of motor units that have mechanically distinct actions. It has been suggested that the nervous system may actually encode more muscle synergies than muscles, but that only a subset of the entire synergy library is used in any given task (Chiel et al., 2009). Such a scheme clearly does not alleviate redundancy, and although it could conceivably simplify control within the context of a given task, it would require an additional control process to select appropriate synergies for each task. It would also appear to be impossible to generate testable hypotheses regarding the existence of an unlimited number of unrealized synergies.

If the nervous system does not control movements through a limited number of synergies, then how does it decide which of many good-enough (i.e., redundant) motor programs to use? Recent modeling work that includes the spinal cord circuitry provides interesting insight into this question. Indeed, a system with a large number of control inputs to a realistic set of interneuronal pathways was found to enable a simple learning algorithm to rapidly converge to physiological solutions (Raphael et al., 2010; Tsianos et al., 2011). Instead of reducing the dimensionality of control signals to assist computation of an unlikely global optimum, the nervous system might take advantage of the high probability of finding good-enough local minima within the high dimensional space of low-level circuitry (Loeb, 2012). A system that learned and stored such motor habits would appear to be limited to the synergies that it happened to have learned, but not as a result of any fundamental mechanism. Such a system might tend to get stuck in motor habits that could become suboptimal if the musculoskeletal system were to change its properties. This is exactly what we found when we applied our virtual biomechanics methodology (de Rugy et al., 2012c) to studies of wrist control in human subjects (de Rugy et al., 2012b).

### Conflict of interest statement

The authors declare that the research was conducted in the absence of any commercial or financial relationships that could be construed as a potential conflict of interest.

## References

[B1] Allen J. L. Neptune R. R. (2012). Three-dimensional modular control of human walking. J. Biomech. 45, 2157–2163 10.1016/j.jbiomech.2012.05.037 22727468 PMC3405171

[B2] Andersen R. A. Essick G. K. Siegel R. M. (1985). Encoding of spatial location by posterior parietal neurons. Science 230, 456–458 10.1126/science.4048942 4048942

[B3] Baraduc P. Guigon E. Burnod Y. (2001). Recoding arm position to learn visuomotor transformations. Cereb. Cortex 11, 906–917 10.1093/cercor/11.10.906 11549613

[B4] Berniker M. Jarc A. Bizzi E. Tresch M. C. (2009). Simplified and effective motor control based on muscle synergies to exploit musculoskeletal dynamics. Proc. Natl. Acad. Sci. U.S.A. 106, 7601–7606 10.1073/pnas.0901512106 19380738 PMC2678607

[B5] Bernstein N. A. (1967). The Coordination and Regulation of Movements. Oxford: Pergamon Press

[B6] Brown I. E. Cheng E. J. Loeb G. E. (1999). Measured and modeled properties of mammalian skeletal muscle. II. The effects of stimulus frequency on force-length and force-velocity relationships. J. Muscle Res. Cell Motil. 20, 627–643 10672511 10.1023/a:1005585030764

[B7] Burkholder T. J. van Antwerp K. W. (2012). Practical limits on muscle synergy identification by non-negative matrix factorization in systems with mechanical constraints. Med. Biol. Eng. Comput. 51, 187–196 10.1007/s11517-012-0983-8 23124815 PMC3582774

[B8] Chanaud C. M. Pratt C. A. Loeb G. E. (1991a). Functionally complex muscles of the cat hindlimb. II. Mechanical and architectural heterogenity within the biceps femoris. Exp. Brain Res. 85, 257–270 1893979 10.1007/BF00229405

[B9] Chanaud C. M. Pratt C. A. Loeb G. E. (1991b). Functionally complex muscles of the cat hindlimb. V. The roles of histochemical fiber-type regionalization and mechanical heterogeneity in differential muscle activation. Exp. Brain Res. 85, 300–313 1832646 10.1007/BF00229408

[B10] Cheng E. J. Loeb G. E. (2008). On the use of musculoskeletal models to interpret motor control strategies from performance data. J. Neural Eng. 5, 232–253 10.1088/1741-2560/5/2/014 18506076

[B11] Cheung V. C. d'Avella A. Tresch M. C. Bizzi E. (2005). Central and sensory contributions to the activation and organization of muscle synergies during natural motor behaviors. J. Neurosci. 25, 6419–6434 10.1523/JNEUROSCI.4904-04.2005 16000633 PMC6725265

[B12] Chhabra M. Jacobs R. A. (2006). Properties of synergies arising from a theory of optimal motor behavior. Neural Comput. 18, 2320–2342 10.1162/neco.2006.18.10.2320 16907628

[B13] Chiel H. J. Ting L. H. Ekeberg O. Hartmann M. J. (2009). The brain in its body: motor control and sensing in a biomechanical context. J. Neurosci. 29, 12807–12814 10.1523/JNEUROSCI.3338-09.2009 19828793 PMC2794418

[B14] Clark D. J. Ting L. H. Zajac F. E. Neptune R. R. Kautz S. A. (2010). Merging of healthy motor modules predicts reduced locomotor performance and muscle coordination complexity post-stroke. J. Neurophysiol. 103, 844–857 10.1152/jn.00825.2009 20007501 PMC2822696

[B15] d'Avella A. Fernandez L. Portone A. Lacquaniti F. (2008). Modulation of phasic and tonic muscle synergies with reaching direction and speed. J. Neurophysiol. 100, 1433–1454 10.1152/jn.01377.2007 18596190

[B16] d'Avella A. Portone A. Fernandez L. Lacquaniti F. (2006). Control of fast-reaching movements by muscle synergy combinations. J. Neurosci. 26, 7791–7810 10.1523/JNEUROSCI.0830-06.2006 16870725 PMC6674215

[B17] d'Avella A. Saltiel P. Bizzi E. (2003). Combinations of muscle synergies in the construction of a natural motor behavior. Nat. Neurosci. 6, 300–308 10.1038/nn1010 12563264

[B18] Davoodi R. Brown I. E. Loeb G. E. (2003). Advanced modeling environment for developing and testing FES control systems. Med. Eng. Phys. 25, 3–9 12485781 10.1016/s1350-4533(02)00039-5

[B19] Davoodi R. Brown I. E. Todorov E. Loeb G. E. (2002a). A biomechanical model of the partially paralyzed human arm, in 7th Annual Conference IFESS (Brisbane, QLD).

[B20] Davoodi R. Kleiman D. Murakata T. Loeb G. E. (2002b). A biomechanical model of the elbow, forearm and wrist, in The 4th World Congress of Biomechanics (Calgary, AB: University of Calgary).

[B21] de Rugy A. (2010). Generalization of visuomotor adaptation to different muscles is less efficient: experiment and model. Hum. Mov. Sci. 29, 684–700 10.1016/j.humov.2010.01.008 20674052

[B22] de Rugy A. Davoodi R. Carroll T. J. (2012a). Changes in wrist muscle activity with forearm posture: implications for the study of sensorimotor transformations. J. Neurophysiol. 108, 2884–2895 10.1152/jn.00130.2012 22972965

[B23] de Rugy A. Loeb G. E. Carroll T. J. (2012b). Muscle coordination is habitual rather than optimal. J. Neurosci. 32, 7384–7391 10.1523/JNEUROSCI.5792-11.2012 22623684 PMC6622296

[B24] de Rugy A. Loeb G. E. Carroll T. J. (2012c). Virtual biomechanics: a new method for online reconstruction of force from EMG recordings. J. Neurophysiol. 108, 3333–3341 10.1152/jn.00714.2012 23019006

[B25] de Rugy A. Hinder M. R. Woolley D. G. Carson R. G. (2009). The synergistic organization of muscle recruitment constrains visuomotor adaptation. J. Neurophysiol. 101, 2263–2269 10.1152/jn.90898.2008 19225174

[B26] de Rugy A. Riek S. Oytam Y. Carroll T. J. Davoodi R. Carson R. G. (2008). Neuromuscular and biomechanical factors codetermine the solution to motor redundancy in rhythmic multijoint arm movement. Exp. Brain Res. 189, 421–434 10.1007/s00221-008-1437-2 18545990

[B27] Diedrichsen J. Shadmehr R. Ivry R. B. (2010). The coordination of movement: optimal feedback control and beyond. Trends Cogn. Sci. 14, 31–39 10.1016/j.tics.2009.11.004 20005767 PMC4350769

[B28] Dominici N. Ivanenko Y. P. Cappellini G. d'Avella A. Mondi V. Cicchese M. (2011). Locomotor primitives in newborn babies and their development. Science 334, 997–999 10.1126/science.1210617 22096202

[B29] Fagg A. H. Shah A. Barto A. G. (2002). A computational model of muscle recruitment for wrist movements. J. Neurophysiol. 88, 3348–3358 10.1152/jn.00621.2001 12466451

[B30] Hargrove L. J. Li G. Englehart K. B. Hudgins B. S. (2009). Principal components analysis preprocessing for improved classification accuracies in pattern-recognition-based myoelectric control. IEEE Trans. Biomed. Eng. 56, 1407–1414 10.1109/TBME.2008.2008171 19473932

[B31] Hauschild M. Mulliken G. H. Fineman I. Loeb G. E. Andersen R. A. (2012). Cognitive signals for brain-machine interfaces in posterior parietal cortex include continuous 3D trajectory commands. Proc. Natl. Acad. Sci. U.S.A. 109, 17075–17080 10.1073/pnas.1215092109 23027946 PMC3479517

[B32] Hug F. (2011). Can muscle coordination be precisely studied by surface electromyography? J. Electromyogr. Kinesiol. 21, 1–12 10.1016/j.jelekin.2010.08.009 20869882

[B33] Jiang N. Englehart K. B. Parker P. A. (2009). Extracting simultaneous and proportional neural control information for multiple-DOF prostheses from the surface electromyographic signal. IEEE Trans. Biomed. Eng. 56, 1070–1080 10.1109/TBME.2008.2007967 19272889

[B34] Kargo W. J. Ramakrishnan A. Hart C. B. Rome L. C. Giszter S. F. (2010). A simple experimentally based model using proprioceptive regulation of motor primitives captures adjusted trajectory formation in spinal frogs. J. Neurophysiol. 103, 573–590 10.1152/jn.01054.2007 19657082 PMC2807239

[B35] Kuiken T. A. Li G. Lock B. A. Lipschutz R. D. Miller L. A. Stubblefield K. A. (2009). Targeted muscle reinnervation for real-time myoelectric control of multifunction artificial arms. JAMA 301, 619–628 10.1001/jama.2009.116 19211469 PMC3036162

[B36] Kuiken T. A. Miller L. A. Lipschutz R. D. Lock B. A. Stubblefield K. Marasco P. D. (2007). Targeted reinnervation for enhanced prosthetic arm function in a woman with a proximal amputation: a case study. Lancet 369, 371–380 10.1016/S0140-6736(07)60193-7 17276777

[B37] Lee D. D. Seung H. S. (2001). Algorithms for non-negative matrix factorization. Adv. Nural Info. Proc. Syst. 13, 556–562

[B38] Lockhart D. B. Ting L. H. (2007). Optimal sensorimotor transformations for balance. Nat. Neurosci. 10, 1329–1336 10.1038/nn1986 17873869

[B39] Loeb G. E. (1993). The distal hindlimb musculature of the cat: interanimal variability of locomotor activity and cutaneous reflexes. Exp. Brain Res. 96, 125–140 8243575 10.1007/BF00230446

[B40] Loeb G. E. (1999). Asymmetry of hindlimb muscle activity and cutaneous reflexes after tendon transfers in kittens. J. Neurophysiol. 82, 3392–3405 10601470 10.1152/jn.1999.82.6.3392

[B41] Loeb G. E. (2000). Overcomplete musculature or underspecified tasks? Motor Control 4, 81–83 discussion: 97–116. 10675814 10.1123/mcj.4.1.81

[B42] Loeb G. E. (2012). Optimal isn't good enough. Biol. Cybern. 106, 757–765 10.1007/s00422-012-0514-6 22895830

[B43] Loeb G. E. Davoodi R. (2005). The functional reanimation of paralyzed limbs. IEEE Eng. Med. Biol. Mag. 24, 45–51 16248116 10.1109/memb.2005.1511499

[B44] Loeb G. E. Gans C. (1986). Electromyography for Experimentalists. Chicago, IL: University of Chicago Press

[B45] Macpherson J. M. (1991). How flexible are muscle synergies? in Motor Control: Concepts and Issues, eds Freund H.-J. Humphrey D. R. (New York, NY: Wiley Press), 33–47

[B46] Mardia K. V. Kent J. T. Bibby J. M. (1979). Multivariate Analysis. London: Academic

[B47] McKay J. L. Ting L. H. (2008). Functional muscle synergies constrain force production during postural tasks. J. Biomech. 41, 299–306 10.1016/j.jbiomech.2007.09.012 17980370 PMC4350792

[B48] McKay J. L. Ting L. H. (2012). Optimization of muscle activity for task-level goals predicts complex changes in limb forces across biomechanical contexts. PLoS Comput. Biol. 8:e1002465 10.1371/journal.pcbi.1002465 22511857 PMC3325175

[B49] Muceli S. Boye A. T. d'Avella A. Farina D. (2010). Identifying representative synergy matrices for describing muscular activation patterns during multidirectional reaching in the horizontal plane. J. Neurophysiol. 103, 1532–1542 10.1152/jn.00559.2009 20071634

[B50] Musallam S. Corneil B. D. Greger B. Scherberger H. Andersen R. A. (2004). Cognitive control signals for neural prosthetics. Science 305, 258–262 10.1126/science.1097938 15247483

[B51] Neptune R. R. Clark D. J. Kautz S. A. (2009). Modular control of human walking: a simulation study. J. Biomech. 42, 1282–1287 10.1016/j.jbiomech.2009.03.009 19394023 PMC2696580

[B52] Oliveira A. S. Gizzi L. Kersting U. G. Farina D. (2012). Modular organization of balance control following perturbations during walking. J. Neurophysiol. 108, 1895–1906 10.1152/jn.00217.2012 22773783

[B53] Parker P. Englehart K. Hudgins B. (2006). Myoelectric signal processing for control of powered limb prostheses. J. Electromyogr. Kinesiol. 16, 541–548 10.1016/j.jelekin.2006.08.006 17045489

[B54] Pouget A. Snyder L. H. (2000). Computational approaches to sensorimotor transformations. Nat. Neurosci. 3 Suppl., 1192–1198 10.1038/81469 11127837

[B55] Pratt C. A. Chanaud C. M. Loeb G. E. (1991). Functionally complex muscles of the cat hindlimb. IV. Intramuscular distribution of movement command signals and cutaneous reflexes in broad, bifunctional thigh muscles. Exp. Brain Res. 85, 281–299 1893981 10.1007/BF00229407

[B56] Pratt C. A. Loeb G. E. (1991). Functionally complex muscles of the cat hindlimb. I. Patterns of activation across sartorius. Exp. Brain Res. 85, 243–256 1893978 10.1007/BF00229404

[B57] Raphael G. Tsianos G. A. Loeb G. E. (2010). Spinal-like regulator facilitates control of a two-degree-of-freedom wrist. J. Neurosci. 30, 9431–9444 10.1523/JNEUROSCI.5537-09.2010 20631172 PMC6632449

[B58] Roh J. Rymer W. Z. Beer R. F. (2012). Robustness of muscle synergies underlying three-dimensional force generation at the hand in healthy humans. J. Neurophysiol. 107, 2123–2142 10.1152/jn.00173.2011 22279190 PMC3331600

[B59] Salinas E. Abbott L. F. (1995). Transfer of coded information from sensory to motor networks. J. Neurosci. 15, 6461–6474 7472409 10.1523/JNEUROSCI.15-10-06461.1995 PMC6578023

[B60a] Selvanayagam V. S. Riek S. Carroll T. J. (2011). Early neural responses to strength training. J. Appl. Physiol. 111, 367–375 10.1152/japplphysiol.00064.2011 21551014

[B60] Staudenmann D. Roeleveld K. Stegeman D. F. Van D. J. H. (2010). Methodological aspects of SEMG recordings for force estimation—a tutorial and review. J. Electromyogr. Kinesiol. 20, 375–387 10.1016/j.jelekin.2009.08.005 19758823

[B61] Ting L. H. Chvatal S. A. (2010). Decomposing muscle activity in motor tasks: methods and interpretation, in Motor Control: Theories, Experiments, and Applications, eds Latash M. L. Danion F. (New York, NY: Oxford University Press), 102–138

[B62] Ting L. H. McKay J. L. (2007). Neuromechanics of muscle synergies for posture and movement. Curr. Opin. Neurobiol. 17, 622–628 10.1016/j.conb.2008.01.002 18304801 PMC4350235

[B63] Todorov E. (2004). Optimality principles in sensorimotor control. Nat. Neurosci. 7, 907–915 10.1038/nn1309 15332089 PMC1488877

[B64] Todorov E. Li W. Pan X. (2005). From task parameters to motor synergies: a hierarchical framework for approximately-optimal control of redundant manipulators. J. Robot. Syst. 22, 691–710 10.1002/rob.20093 17710121 PMC1945248

[B65] Torres-Oviedo G. Macpherson J. M. Ting L. H. (2006). Muscle synergy organization is robust across a variety of postural perturbations. J. Neurophysiol. 96, 1530–1546 10.1152/jn.00810.2005 16775203

[B66] Tresch M. C. Jarc A. (2009). The case for and against muscle synergies. Curr. Opin. Neurobiol. 19, 601–607 10.1016/j.conb.2009.09.002 19828310 PMC2818278

[B67] Tsianos G. A. Raphael G. Loeb G. E. (2011). Modeling the potentiality of spinal-like circuitry for stabilization of a planar arm system. Prog. Brain Res. 194, 203–213 10.1016/B978-0-444-53815-4.00006-6 21867805

[B68] Valero-Cuevas F. J. Hoffmann H. Kurse M. U. Kutch J. J. Theodorou E. A. (2009). Computational models for neuromuscular function. IEEE Rev. Biomed. Eng. 2, 110–135 10.1109/RBME.2009.2034981 21687779 PMC3116649

